# Copulatory behaviour in the Bonelli´s Eagle: Assessing the paternity assurance hypothesis

**DOI:** 10.1371/journal.pone.0217175

**Published:** 2019-05-21

**Authors:** José E. Martínez, Iñigo Zuberogoitia, José M. Escarabajal, Ginés J. Gómez, José F. Calvo, Antoni Margalida

**Affiliations:** 1 Bonelli´s Eagle Study and Conservation Group, Murcia, Spain; 2 Departamento de Ecología e Hidrología, Universidad de Murcia, Campus de Espinardo, Murcia, Spain; 3 Estudios Medioambientales Icarus S.L. C/ San Vicente, Bilbao, Bizkaia, Spain; 4 Institute for Game and Wildlife Research, IREC (CSIC-UCLM-JCCM), Ciudad Real, Spain; 5 Division of Conservation Biology, Institute of Ecology and Evolution, University of Bern, Bern, Switzerland; University of Tulsa, UNITED STATES

## Abstract

We examined copulatory behaviour in the Bonelli´s Eagle (*Aquila fasciata*) at nesting sites in the eastern zone of the Baetic Cordillera, southern Spain, between 2010 and 2012. We observed the copulatory behaviour of 15 pairs during the pre-laying period. Bonelli´s Eagles commenced sexual activity ca. 69 days before egg-laying. Ninety-six percent of mounting attempts were successful. Bonelli´s Eagle pairs averaged 99.8 copulation attempts per clutch, with an average copulation frequency of 0.86 copulation attempts per day. Pairs displayed a daily bimodal pattern of copulation activity, with copulations occurring most frequently in the evening. We used our data to test three predictions with regard to the paternity assurance hypothesis. Prediction 1, that within-pair copulations increase with local breeding density, was rejected because our models showed no evidence for it. Prediction 2, that within-pair copulations increase during the female fertile period, was marginally supported. Finally, Prediction 3, that mate attendance increases during the female fertile period, was also rejected because mate-guarding did not increase as the fertile period approached. However, mate-guarding was positively correlated with within-pair copulation frequency. Moderate copulation rates compared to other raptors and the absence of mate-guarding suggest that, in the study area, Bonelli´s Eagles exhibit only partially adaptive behaviour to assure their paternity. A possible explanation could be related to the low number of extra-pair encounters observed (opportunities for which appear to be rare), although the gradual increase in within-pair copulations during the female fertile period is consistent with the sperm competition hypothesis. The results are discussed based on the signalling hypothesis, which proposes that raptors signal territory ownership to conspecifics, and possibly to other raptor species, by copulating frequently and conspicuously in the defended nesting area.

## Introduction

Copulatory behaviour and the frequency of mounting attempts is extremely variable in vertebrates [1]. In most taxa, copulations occur several times before the female fertile period, during each breeding season [2, 3, 4], whereas in other taxa, such as birds of prey and dolphins, copulations may occur many times over long periods, both inside and outside of the female´s fertile period [1]. However, to date, the factors determining this aspect of mating behaviour are not well understood [1, 5].

Some raptor species perform frequent copulations outside the fertile period, which presumably serve functions other than fertilization such as mate assessment, pair bonding [1, 5] or territorial signaling [6, 7]. However, in most species courtship and copulation start within the two months before egg laying, although the frequency of copulations reaches its maximum close to the laying date [3]. Many copulations performed by raptors at the beginning of the courtship period are “fake”, and serve mainly to inform neighbours and potential intruders of the presence of a territorial couple [6]. While the males of some raptor species share parental care with their mates, they will take opportunities to fertilize mated females and thus parasitize the parental investment of other males [2]. Extra-pair copulations (EPCs) have been documented in a wide of range of bird species, and the frequency of within-pair copulations (WPCs) represents a preventive strategy to reduce the likelihood of extra-pair fertilizations being successful in fertilizing eggs [2, 3, 8]. Sexual selection would favour male traits that both ensure their own paternity and increase their reproductive success through extra-pair matings [9, 10].

Paternity assurance is critical to male fitness, particularly in bird species in which females frequently perform EPCs and males contribute to costly parental care [11]. In response to fitness loss due to cuckoldry, males have evolved several preventative mechanisms to reduce the risk of paternity loss. In monogamous raptors, mate-guarding and repeated WPCs comprise the main strategies for paternity assurance [2, 12]. When mate-guarding, males follow their females during the fertile period closely and prevent copulations with competitor males [2, 13, 14, 15]; for example the aggressive behaviour shown by territorial males towards male intruders [16]. However, ecological constraints can reduce the efficiency of such preventive strategies [17], for example in colonially breeding species or in territorial species where males have to compromise between leaving the breeding site to forage for themselves and their mate and staying close to the female to assure paternity [18, 19]. An alternative paternity assurance mechanism is to copulate at very high rates. Males that reduce the risk of cuckoldry using high copulation rates have increased sperm production capacities [8], increasing the probability that their sperm will be the last present just before fertilization occurs [2], and may lead to females disguising their fertile period in order to avoid EPCs [8].

Our study species, the Bonelli´s Eagle *Aquila fasciata*, is currently considered Endangered in Spain [20] and as of Least Concern worldwide [21]. It is a socially monogamous, territorial raptor, with moderate sexual dimorphism and differing sex roles during the breeding season, as is general in large eagles [22]. Males are the main food providers during incubation and for about 90% of the nestling period, whereas females perform most of the incubation, attend closely to the young and defend the nesting territory [23]. It shows great fidelity to its nest site and surroundings, both during and outside the breeding season, indicating the importance of defending this resource both intra- and interspecifically [24]. It usually hunts singly, but pairs sometimes hunt together [25] sharing their skills [23, 26]. During the males’ foraging trips, females remain alone at the nest-site, where they may sometimes seek EPCs with intruders. However, conspecifics are usually chased from the territory by both sexes; territorial defence is generally carried out by males during the nestling period, while females remain close to their nest [23]. Territorial adults remain together throughout year [23].

Little is known about the copulatory behaviour of the Bonelli´s Eagle; previous studies have compiled some data based on small sample sizes [23, 26, 27]. The main objectives of our study were therefore two-fold: (i) to describe in detail the copulatory behaviour of Bonelli´s Eagles during the pre-laying period; and (ii) to test the adaptive significance of WPCs in relation to paternity assurance. We tested three predictions: (1) WPC frequency increases at high breeding densities; (2) WPC frequency increases during the female fertile period; and (3) mate-guarding at the nesting site and its surroundings increases during the fertile period.

Prediction 1 is that WPC frequency increases during the fertile period at high breeding densities, where risk of EPCs is high [4, 28, 29, 30]. Numerous studies have confirmed this relationship [4, 12, 29, 31], although other investigations have not [5, 32]. Prediction 2, that WPC frequency increases during the fertile period, would be expected because a higher copulation frequency would be more efficient in diluting ejaculates from EPCs at the moment in which the female fertilizes her eggs [1]. Prediction 3 is that mate-guarding increases during the fertile period [1] because males actively guard their paternity by aggressively interacting with extra-pair males who try to copulate with their female mates [2, 33, 34]. By remaining close to the female, the male may deter any extra-pair males from approaching her and may also deter the female from soliciting EPCs [34].

## Material and methods

### Ethics statement

The study was conducted in full compliance with Spanish laws and regulations. Birds were observed from dominant vantage points at a prudent distance to nests in order to avoid disturbances. Birds always seemed unconcerned by the presence of the observer. Because our study was observational, when no animal experiments were carried out, and average monitoring distances were further than the minimum distance allowed by environmental regulations to observe a nest of this species, no other permissions were required.

### Study area

Field work was carried out during 2010–2012 in a large area located in the south of the Murcia Region, east of Almería province in southeastern Spain (coordinates 37° 43´ N, 1° 38´ O). The study area is a mountainous region of 1,200 km^2^ with a typically semiarid Mediterranean climate (mean annual rainfall of 300 mm and an annual average temperature of 17°C, see [35]. Autumn and winter are characterized by moderate temperatures and low precipitation. Our study area includes part of the eastern zone of the Baetic Cordillera where the Bonelli´s Eagle population density is low [36]. The average altitude of Bonelli’s Eagle nesting sites is 432 m (range 100–650 m).

Data were collected during two consecutive breeding seasons (2010–2011 and 2011–2012). Observations were performed between October (before courtship onset) and February (when all females were already incubating) on seven pairs during the first breeding season and eight pairs during the second (N = 15 pairs). We assumed a fertile period of < 14 days before egg-laying, taking day 0 as the egg-laying date [37]. Laying onset was determined visually, while checking female behaviour on the nest.

The pairs were chosen on the basis of their visibility from nearby cliffs [19]. Eagles were monitored using 10x binoculars and 20–60x telescopes at a prudent distance of 500–800 m from the cliff face where the nests were located, to avoid observer disturbance. Individuals were sexed visually by size comparisons based on the species’ sexual dimorphism [38, 39] and by the positions adopted during mounting attempts. Observations were performed once every week per pair. On each observation day, we monitored the eagles´ behaviour through the daylight hours from 06:00–18:00 (before sunrise–after sunset). The observations were ended following egg-laying. In total, we performed 1,860 hours of observations during the pre-laying period and a further 164 hours during the incubation period, in order to record post-laying sexual activity. We recorded the identity of the pair; whether the copulation was successful or not (i.e., whether cloacal contact was achieved during mounting); the duration of copulation attempts measured with a stopwatch (in seconds); the time of each mating event and its location (nest, roost, perches); the length of time that eagles were present at nest-sites (in mins); and any other activities related to copulatory behaviour [40]. Additional details of the variables are described in S1 Data. We defined territorial intrusions as the entry of another Bonelli´s Eagle into the breeding territory of the target pair (i.e., within a distance of ca. 800–1000 m), which usually resulted in territorial defence or protective displays by pair members. We also recorded males guarding mates (hereafter, mate-guarding) as the time (in mins) that the male and female of a pair spent within at least 100 m of each another, either flying, at the nest-site, or perched [1]. In addition, we recorded mate absence as the time (in mins) that the male or female of a pair spent foraging away from the nest-site. We calculated the local breeding density as the number of other nesting pairs within a 10 km radius of the monitored nest.

### Data analysis

The relative frequency of copulation was estimated as the number of attempts hr^-1^ per monitoring day. The frequencies recorded were combined into 7-day periods dating backwards from egg-laying (taken as day 0).

In order to test Prediction 1, we analyzed whether the copulation frequency (WPC, Table 1) could be predicted by the density of breeding territories (DENS). We tested Prediction 2 by considering WPC as a response variable, WEEK (time period) as a covariable and PFER (pre-fertile and fertile period) as the fixed factor. We built generalized linear mixed models (GLMM) fitted with the Laplace approximation [41] and using WPC as the dependent variable under a Poisson distribution (log-link function). Finally, we tested Prediction 3 by applying a second GLMM to analyse whether mate vigilance was influenced by the fertile period. We used the mate-guarding time (MAFE, Table 1) as the response variable under a normal distribution and PFER as the predictive variable. To account for possible effects of correlation factors in the data, male identity was included as a random factor in all cases. All statistical analyses were performed with R 3.2.2 [42]. The GLMM was analysed using the “lme4” and ‘car’ packages [43]. Mean values are reported with their standard errors. Statistical significance was set at *P* < 0.05. Additional details of the models are described in S2 Data.

**Table 1:** Variables used in models to analyse within-pair copulation attempts and intensity of mate-guarding in Bonelli´s Eagles.

Acronym	Definition
DENS	Number of overlapping home ranges of neighbouring breeding pairs within a 10 km radius of the target nest. A discrete quantitative variable (range: 0–7).
WEEK	Number of weeks before egg-laying until the week of the onset of laying. A discrete quantitative variable (range: 16–1).
MAFEH	Time spent mate-guarding by the male within the nest surroundings, per fieldwork day (in hours).
WPC	Frequency of within-pair copulations. A discrete quantitative variable (range: 0–5).
PFER	Time periods. A categorical variable with two classes: pre-fertile and fertile.

## Results

### Timing and frequency of copulation attempts

We observed a total of 155 copulation attempts, of which 144 (92.9%) occurred during the pre-laying period; 139 copulations (96.53%) were successful. The mean duration of successful copulations was 12.09 ± 3.90 s (range: 4.6–31.41 s, N = 150). We recorded 11 copulation attempts after laying. Copulation attempts took place on cliffs, 251.63 ± 305.86 m (range: 10–2,436 m) away from the nest. Most copulations took place at the pairs’ most frequently used perching sites, but 31 (20%) occurred at roost sites. The mountings observed at roost sites took place at dawn and dusk.

**Fig 1:**
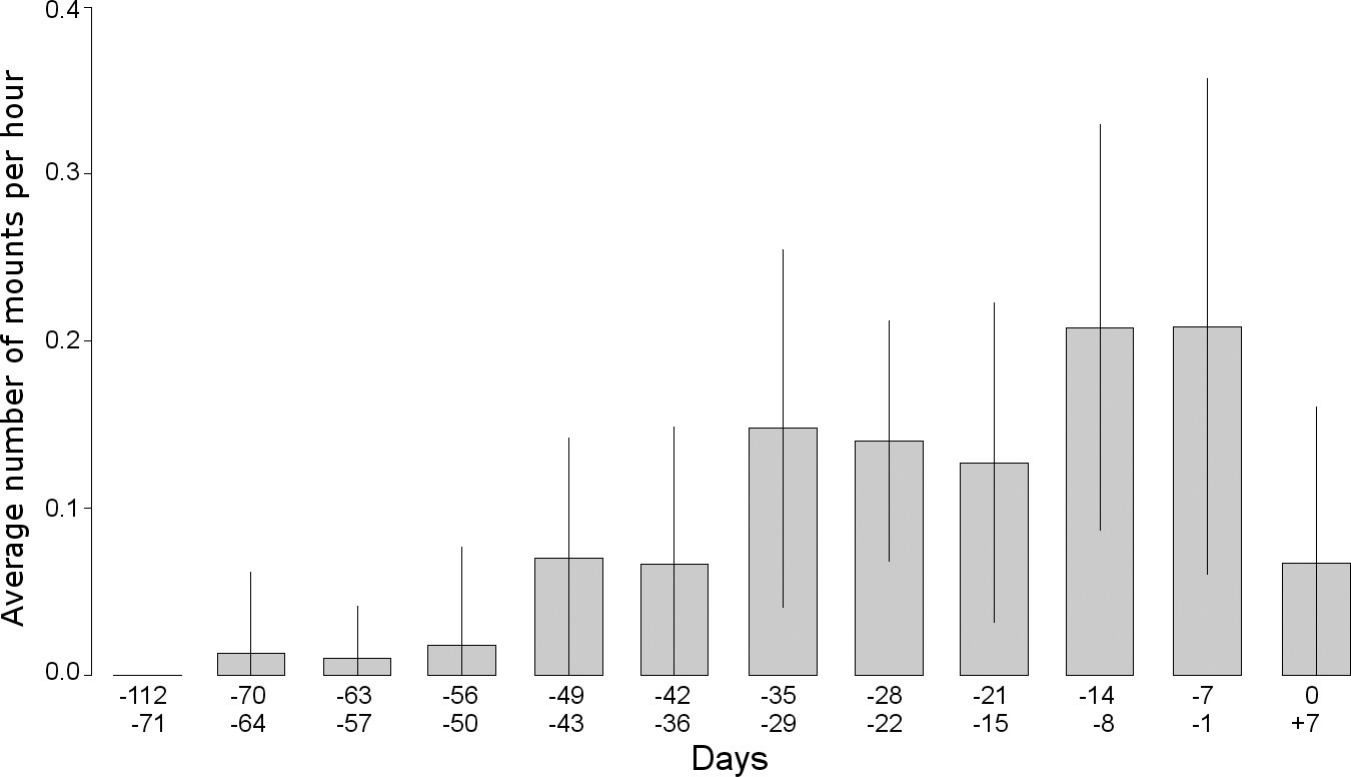
Temporal patterns of mounting in fifteen pairs of Bonelli´s Eagles calculated weekly from before egg-laying until the onset of laying (0 = day of egg-laying). The interval (-112/-71 days) comprises the consecutive six week period before egg-laying (from the 11^th^ to the 16^th^ week). Vertical lines represent the 95% confidence intervals.

The first copulation attempts of a pair were detected between 69 and 29 days (41.06 ± 13.59) before egg-laying. After the first copulation, pairs progressively increased their sexual activity (Fig 1) with two peaks of copulation attempts between 14–8 days before egg-laying (0.20 ± 0.12 copulation attempts h^-1^) and 7–1 days before egg-laying (0.21 ± 0.14 copulation attempts h^-1^), declining thereafter to 0.06 ± 0.09 copulation attempts h^-1^ during days 0 to + 7. The maximum number of behaviourally successful copulations observed in a single day was five; twice in two of the pairs (on days -7 and -1, and days -14 and—8, respectively), and four in two other pairs (on days -14 and -8). The minimum time interval observed between two behaviourally successful copulations was 10 min (day -12 at 15:54 and 16:04). All copulation attempts were accompanied by low intensity male vocalisations.

**Fig 2:**
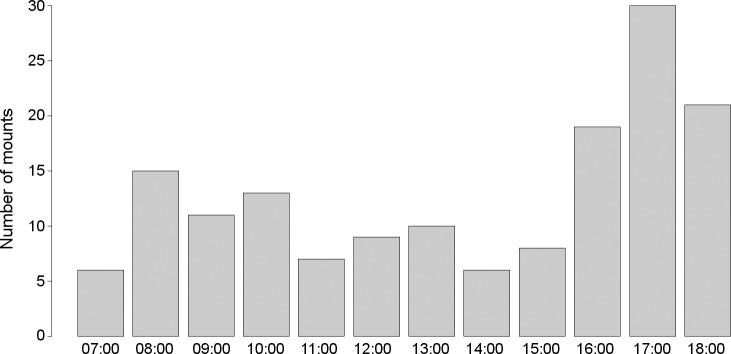
Daily pattern of mounting (number hr^-1^ of observation) of 15 pairs of Bonelli´s Eagles during the 10 weeks prior to egg-laying (days –70 to +7, N = 155 copulation attempts).

The daily copulatory behaviour showed a bimodal pattern (Fig 2), with a first peak in the morning (07:00–10:00 h) and another, more marked peak, in the afternoon (15:00–18:00 h). The average copulation rate was 0.86 ± 0.46 copulation attempts per day (range 0.18–1.60, N = 15 pairs). The number of copulations per day during the female´s fertile period was 1.71 ± 1.48 (range 0–5). Assuming 11 h available for daylight activity during October–February, we estimated a mean of 99.79 ± 52.45 copulation attempts per clutch (range 21.82–182.64).

No EPC attempts were observed, and numbers of territorial intrusions were low (*N* = 6; 1 juvenile, 1 immature, 3 subadults and 1 female adult). Territorial intrusions resulted in agonistic interactions, except in one case where both adults were absent from the territory. Three intruders were chased by the pair, one only by the male, and another only by the female.

### Testing the paternity assurance hypothesis

**Fig 3:**
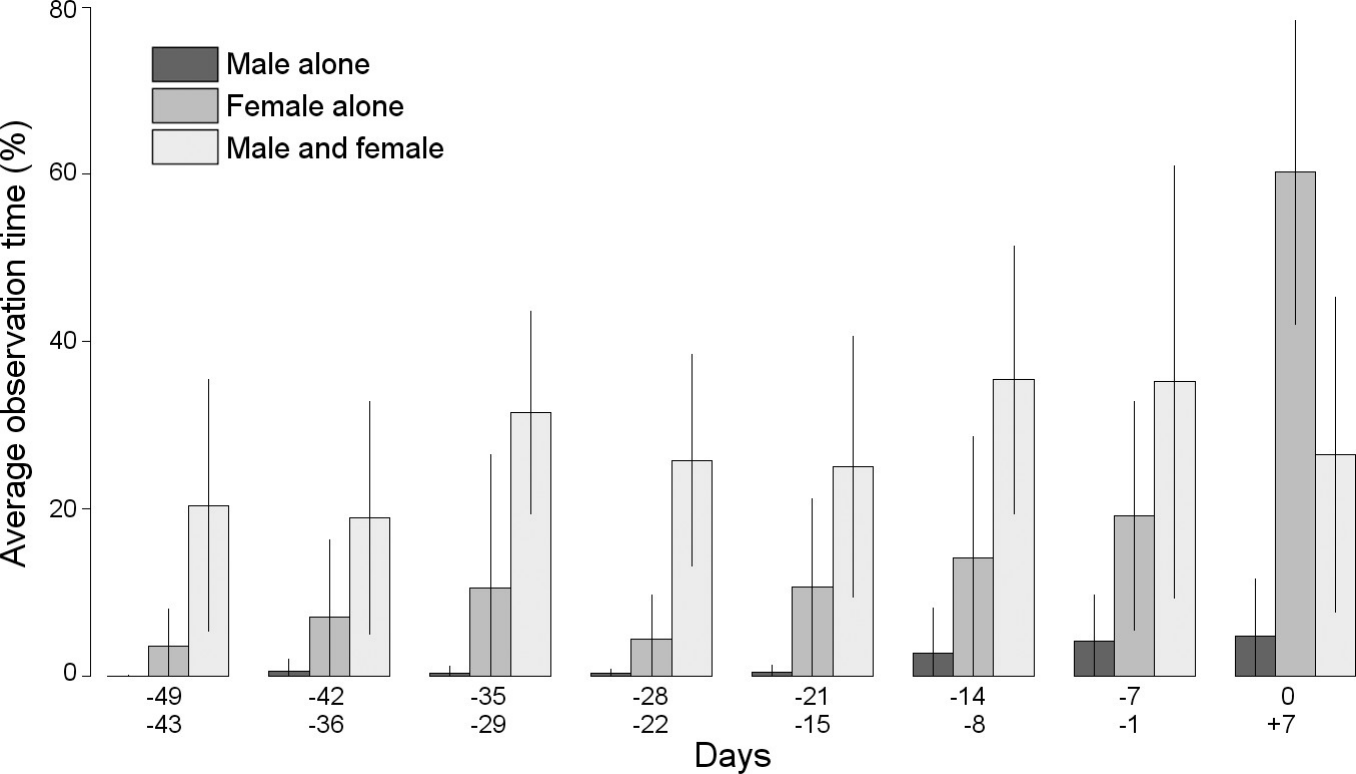
Average observation time (%) of females and males together at nesting sites, for the 15 pairs, corresponding to the period between days -49 and +7. Vertical lines represent the 95% confidence intervals.

Our models rejected Prediction 1, that the density of breeding pairs would affect WPC (Table 2). With respect to Prediction 2, the WPC frequency increased steadily as egg-laying approached, and throughout the fertile period. Lastly, our data supported Prediction 3; mate-guarding was positively correlated with WPC frequency (Table 3; Fig 3).

**Table 2:** Results of generalized linear mixed models for testing Predictions 1 and 2: The copulation frequency (WPC) increased regularly throughout the pre-laying period, using: the density (DENS), expressed as the number of nesting pairs within a 10 km radius of the monitored nest; week (WEEK), expressed as the number of weeks before egg-laying; the time spent guarding the partner within the nest surroundings (MAFEH), using the time that the adults remained together in the territory, expressed as the time that the male spent mate-guarding in the territory; and the fertile period (PFER). All as predictor variables. Significant relationships are shown in bold type. The values for the *z* statistics are also shown.

		Estimate	SE	*z*-value	*P*
Predictions 1 and 2	Intercept	-0.39401	0.27206	-1.448	0.1476
	DENS	0.06077	0.04947	1.228	0.2193
	WEEK	0.22699	0.03763	6.032	**<0.001**
	MAFEH	0.22099	0.04484	4.928	**<0.001**
	PFER	0.64844	0.25679	2.525	**0.011**

**Table 3:** Results of GLIMMs for testing Prediction 3: the time that the male spent mate-guarding (MAFEH) was related to the within-pair copulation rate (WPC). Significant relationships are shown in bold type. The values for the *t* statistics are also shown.

		Estimate	SE	*t*-value	*P*
Prediction 3	Intercept	2.417	0.438	5.511	
	DENS	0.054	0.112	0.480	0.630
	WEEK	0.061	0.033	1.839	0.065
	WPC	0.571	0.112	5.071	**<0.001**
	PFER	-0.351	0.341	-1.029	0.303

## Discussion

Our study shows that Bonelli´s Eagles copulate over a long period (69 days) and at a high rate (99.79 copulations attempts per clutch); considered a common pattern in raptors [4]. Previous studies have proposed that the copulation frequency be considered as high whenever it occurs 20 times or more per breeding season, or whenever the daily number of copulations during the female´s fertile period is considerably more than two [2, 8]. However, despite the numerically high copulation rates observed in Bonelli’s Eagle, they are moderate in comparison with other raptors such as Northern Goshawk *Accipiter gentilis*, Montagu´s Harrier *Circus pygargus*, Black Kite *Milvus migrans*, Red Kite *Milvus milvus*, Osprey *Pandion haliaetus* and Bearded Vulture *Gypaetus barbatus* [4, 12, 29, 40, 44, 45], all of which exceed 200 mounts per breeding season [5, 6, 40, 45]. In fact, the copulation rates observed in our study are only higher than those described for Egyptian Vultures: 55 copulation attempts [46] and Griffon Vultures: 71 copulation attempts [19]. However, all of these reported estimates of copulation rate should be considered with caution given that most studies have focused only on the nesting area without considering the number of copulations that take place during foraging movements or at roosts.

In our study, the average proportion of successful copulations (96%) is greater than that found in many raptor species [5, 12, 19, 40, 46, 47]; only in the Northern Goshawk has a higher rate been observed [44]. In the case of the Bonelli´s Eagle, the moderate copulation frequency observed could be compensated for by the high copulation success rate, which may also reduce the number of EPC attempts [19].

Previous studies estimated the maximum WPC rate for Bonelli´s Eagle as 3 mounts/day during the weeks preceding laying [23, 26, 27]; a rate significantly lower than that obtained in our study (5 mounts/day). Such differences may be due to differences in the surveillance effort; we detected a significant proportion (20%) of copulations at dawn and dusk at the roosting sites in poor light, which makes them difficult to observe. These inconspicuous copulations would not have been detected if the observations began after dawn and/or if observations ended when the birds return to the roost, at dusk. In future studies it will be important to widen the observation period as much as possible in order to increase the probability of detecting crepuscular copulation attempts. The daily pattern of copulation frequency, with one peak early in the morning and the other, more relevant, at the end of the day, may be explained by the foraging ecology of Bonelli´s Eagles and the risk of EPCs [19, 40]. Before dawn, we often observed Bonelli's Eagles copulating at the roost sites, before both sexes begin their daily activity around the nest, where they often copulated again before going foraging during the middle of the day. As has been suggested for other Mediterranean eagles [48], Bonelli´s Eagles probably use the thermal uplifts during the middle of the day to make long-distance movements outward from the breeding sites to foraging areas. Bonelli´s Eagles copulate again in the evening after meeting again after foraging, consistent with the idea that it is advantageous for males to acquire the last copulations of the day with their mates [40, 49].

### Frequency and density of within-pair copulations

Many bird species copulate frequently, probably as a means to ensure paternity [2, 3]. In raptors, close proximity to neighbours is associated with an increased risk of EPCs, and consequently the intensity of EPC preventive mechanisms increases with local breeding density [4, 29, 30]. Accordingly, previous studies have found positive relationships between copulation frequency and nesting density [4, 12, 13, 28, 29, 31]. However, our results did not support this hypothesis (Prediction 1) and, at least in our study area, the WPC frequency of Bonelli’s Eagles was not related to the risk of loss of paternity via EPCs [40]. This result indicates males’ perceived low risk of EPCs as a result of the low level of territorial intrusions observed during our intensive field observations (six probable intrusion attempts in only 33% of the monitored nests).

Our results showed that the frequency of WPCs increased as laying approached and during the presumed female fertile period [3, 12, 30, 37, 50, 51], that is the period when Bonelli´s Eagle pairs spent more time together at their nests. This could explain the significant positive relation between WPC frequency and mate-guarding [46]. Crowe et al. [15] showed that frequent copulations just before the start of egg-laying is an adaptation to decrease the risk of being cuckolded (e.g. sperm competition). Consequently, our results may support Prediction 2 and are consistent with the hypothesis that sperm competition enhances paternity assurance. However, the WPC frequency and its positive association with the other explicative variables does not seem to fit sperm completion related explanations, as described in previous studies on the risk of paternity loss at nest sites and their surroundings in other raptors [13, 19]. A possible explanation may be that high copulation rates in the surroundings of the nest could compensate for the risk of EPCs away from nesting sites as a consequence of their foraging strategies (e.g. when the female forages alone during the pre-laying period), as has been documented for Bearded Vultures [40]. Therefore, males could adapt their copulation frequencies to both their own movements and those of their mates [40, 44]. In our study, Bonelli´s Eagles spent 66% of each day ranging over their foraging areas; although information on foraging movements as a pair during the pre-laying period are currently scarce [24, 52, 53, 54]. However, recent high-resolution satellite telemetry monitoring revealed that both sexes spend a large part of each day ranging together over their foraging areas during the period preceding egg-laying (Perona & López-López pers. comm.). As a consequence, the risk of EPCs must be low at these times, and consequently low extra-pair paternity rates should be expected.

### Mate-guarding, territorial intrusions and extra-pair copulations

The paternity assurance hypothesis predicts that both WPC and EPC frequencies should increase relative to local breeding density [4]. In socially monogamous species, males adopt preventative behaviours, such as mate-guarding, to minimize cuckoldry risk when they perceive risks of EPCs arising from territorial intrusions [2, 55]. The local nesting density is likely to affect preventative behaviour because as spatial proximity increases, so does the likelihood of direct interaction [31]. However, our study does not support this relationship (Prediction 1). Indeed, our results indicate that the time spent mate-guarding is positively correlated with WPC frequency (Prediction 2); copulation frequency is related to the time that the pair spend together at the breeding site [46]. Previous studies showed that males significantly increased their time with females at the nest, mate-guarding, during the fertile period [37, 40, 46]. Nevertheless, our findings showed that Bonelli´s Eagle pairs did not spend significantly more time together in the vicinity of the nest during the presumed fertile period (Fig 3). Our results also showed that male Bonelli´s Eagles did not significantly increase the time spent with females as egg-laying approached [19], further indicating an absence of the mate-guarding strategy (and thus, not supporting Prediction 3), suggesting few behavioural adaptations for paternity assurance, and no consistency with the sperm competition or paternity assurance hypotheses. Moreover, because we did not observe any EPCs, sperm competition is apparently fairly unimportant in our study area.

In summary, our results indicate that Bonelli´s Eagles have a high copulation frequency. However, this behaviour does not seem to be related to the potential risk of paternity loss, which leads us to consider alternative hypotheses to those related to sperm competition; our results do not support the idea that sperm competition is the main cause of the high WPC rate. Some alternative hypotheses could be proposed. The first is that a high copulation frequency during the presumed fertile period could be considered as a signal of a male’s physical condition and fitness, and so play a role in female mate-assessment [56, 57, 58]. A second hypothesis is that high copulation frequency strengthens the pair-bond and is a strategy to reduce the potential risk of losing a partner [59]. Finally, a third hypothesis, known as the signalling hypothesis, proposes that raptors signal territory ownership to potential competitors–both conspecifics and possibly other raptor species–by copulating frequently and conspicuously in the defended nesting area early in the breeding season [6]. This could apply to the case of Bonelli´s Eagle. In our study, individuals vocalized while copulating and the copulations were performed in exposed and prominent places close to the nest site where the presence of intruders is not tolerated. Therefore, this mating behaviour could (i) inform conspecifics and other potential intruders (i.e. Golden Eagle *Aquila chrysaetos* and Peregrine Falcon *Falco peregrinus*) about the nest site occupancy and their breeding status; and (ii) reduce risk of possible dangerous or lethal figths between territory owners and intruders [6]. More research is needed to examine these hypotheses and to better understand the adaptive significance of repeated WPCs in Bonelli's Eagles.

## Supporting information

S1 Data Copulation frequency and behaviour associated with sexual activity in 15 Bonelli´s Eagle pairs in south-eastern Spain. (XLS)

S2 Data Input data for analysis (models). Copulation frequency and behaviour associated with sexual activity in 15 Bonelli´s Eagle pairs in south-eastern Spain.
(XLS)
